# Fertility, gonadal and sexual function in survivors of testicular cancer

**DOI:** 10.1038/sj.bjc.6602677

**Published:** 2005-07-05

**Authors:** R A Huddart, A Norman, C Moynihan, A Horwich, C Parker, E Nicholls, D P Dearnaley

**Affiliations:** 1 Academic Unit of Radiotherapy and Oncology, Institute of Cancer Research and The Royal Marsden NHS Foundation Trust, Downs Road, Sutton, Surrey SM2 5PT, UK; 2 Department of Computing and Information, Institute of Cancer Research and The Royal Marsden NHS Foundation Trust, Downs Road, Sutton, Surrey SM2 5PT, UK

**Keywords:** testicular cancer, quality of life, hormones, fertility

## Abstract

Modern treatments cure most testicular cancer patients, so an important goal is to minimise toxicity. Fertility and sexual functioning are key issues for patients. We have evaluated these outcomes in a cross-sectional study of long-term survivors of testicular cancer. In total, 680 patients treated between 1982 and 1992 completed the EORTC Qly-C-30(qc30) questionnaire, the associated testicular cancer specific module and a general health and fertility questionnaire. Patients have been subdivided according to treatment received: orchidectomy either alone (surveillance, S *n*=169), with chemotherapy (C, *n*=272), radiotherapy (R, *n*=158), or both chemotherapy and radiotherapy (C/RT *n*=81). In the surveillance group, 6% of patients had an elevated LH, 41% an elevated FSH and 11% a low (<10 nmol l^−1^) testosterone. Hormonal function deteriorated with additional treatment, but the effect in general was small. Low testosterone was more common in the C/RT group (37% *P*=0.006), FSH abnormalities were more common after chemotherapy (C 49%, C/RT 71% both *P*<0.005) and LH abnormalities after radiotherapy (11% *P*<0.01) and chemotherapy (10%, *P*<0.001). Baseline hormone data were available for 367 patients. After treatment, compared to baseline, patients receiving chemotherapy had significantly greater elevations of FSH (median rise of 6 (IQR 3–9.25) iu l^−1^ compared to 3 (IQR 1–5) iu l^−1^ for S; *P*<0.001) and a fall (compared to a rise in the surveillance group) in median testosterone levels (−2 (IQR −8.0 to −1.5) *vs* 1.0. (IQR −4.0–4.0) *P*<0.001). Patients with low testosterone (but not elevated FSH) had lower quality of life scores related to sexual functioning on the testicular cancer specific module and lower physical, social and role functioning on the EORTC Qly C-30. Patients with a low testosterone also had higher body mass index and blood pressure. Treatment was associated with reduction in sexual activity and patients receiving chemotherapy had more concerns about fathering children. In total, 207 (30%) patients reported attempting conception of whom 159 (77%) were successful and a further 10 patients were successful after infertility treatment with an overall success rate of 82%. There was a lower overall success rate after chemotherapy (C 71%; CRT 67% compared to S 85% (*P*=0.028)). Elevated FSH levels were associated with reduced fertility (normal FSH 91% *vs* elevated 68% *P*<0.001). In summary, gonadal dysfunction is common in patients with a history of testicular cancer even when managed by orchidectomy alone. Treatment with chemotherapy in particular can result in additional impairment. Gonadal dysfunction reduces quality of life and has an adverse effect on patient health. Most patients retain their fertility, but the risk of infertility is likely to be increased by chemotherapy. Screening for gonadal dysfunction should be considered in the follow-up of testicular cancer survivors.

Testicular cancer is the most common cancer of young men in their 20s and 30s. Since the advent of effective multiagent cisplatinum-based chemotherapy, the majority of patients are cured. This disease occurs during the peak period of reproductive life and at a key time for career and family. As cured testicular patients have a long life expectancy, minimising effects on long-term health and quality of life are important goals. To understand the long-term effects of testicular cancer treatment on fertility, gonadal and sexual function we have surveyed survivors of testicular cancer more than 5 years after diagnosis. These aspects have been studied as part of a broad ranging cross-sectional study of patients treated for testicular cancer between 1982 and 1992 at our institution.

## PATIENTS AND METHODS

Details of the study methodology have been previously reported (Huddart *et al*, 2003). In brief, all UK resident male patients registered between 1982 and 1992 at The Royal Marsden NHS Trust with a diagnosis of germ cell tumour, who had completed cancer treatment more than 5 years previously, were eligible for entry into this study. A total of 1603 patients were registered during this time period. Of these, 200 were considered ineligible (by virtue of overseas residence (163), female sex (3) or recent treatment or additional cancer diagnosis (34)) and 203 had died leaving 1200 patients eligible for recruitment. Of these, 739 patients were recruited with 59 patients declining consent to the study and 402 patients being either lost to follow-up or not seen in the clinic during time-frame of the study (October 1997 and February 1999). The baseline characteristics and treatment received by recruited patients is similar to that of the eligible cohort (Table 1 and see Huddart *et al*, 2003).

Patients gave written consent to the study and completed a general health questionnaire and quality of life form (EORTC QLYC-30) along with the testicular cancer module, which included questions on sexual activity. The health questionnaire asked specific questions regarding fertility following treatment. A clinical review was undertaken and blood was drawn for a range of tests including LH, FSH and testosterone. All biochemical results and blood pressure were notified to the patient's local doctor. Prior to 1990, postorchidectomy, preradiotherapy/chemotherapy gonadal hormone levels were routinely collected. These results form the basis for longitudinal hormone comparisons with matched individual data available on 367 patients. Semen analysis was not performed in this study.

The study was undertaken according to a protocol agreed by the local research ethics committee (Ethics No. 1387). This report will focus on the hormonal function and fertility aspects of the study.

### Statistical methods

Patients registered at the Royal Marsden NHS Trust with a diagnosis of testicular cancer, in the time period 1982–1992, were prospectively identified on the Bob Champion Unit research database prior to initiation of the study (second opinions on management were not included). Eligible patients according to predefined criteria booked in the long-term follow-up clinic in the period from October 1997 to February 1999 were identified and contacted as described in the Materials and methods section.

Information collected at the clinic visit including blood tests and questionnaire information was prospectively entered onto the Bob Champion Unit research database. For all patients with data collected in the clinic, descriptive analysis was performed on all variables and comparisons were made between the four treatment groups. Categorical data were examined using the *χ*^2^ test and continuous variables were compared using the Mann–Whitney nonparametric test unless otherwise stated. Paired data were assessed by the Mann–Whitney or the Wilcoxson tests. Results from the core EORTC Qly C-30 questionaires were analysed according to the standard methods (Fayers *et al*, 2001). Substitutions for missing results were not performed, and incomplete domains were not analysed. The testicular module allowed patients to choose whether or not to answer questions on sexuality. This option was exercised by a variable number of patients according to the question (60–130 per question). Thus, no overall rating is given for this section, but results analysed on a question by question basis.

## RESULTS

### Patient characteristics

Characteristics of the patient cohort are as previously described (Huddart *et al*, 2003) and are summarised in Tables 1 and 2. Of the 739 patients who gave their consent to the the study, 59 have been excluded from this analysis as they had either a second orchidectomy (synchronous *n*=3, metachronous *n*=47) or an absent contralateral testis (*n*=9). Of the remaining cohort of 680, questionnaires were completed by 640. Of these patients, 367 treated prior to 1990 had pretreatment postorchidectomy (baseline) hormone levels. The surveillance group received no treatment except for orchidectomy (*n*=169, 114 with baseline data). This group acts as a reference for derivation of treatment-related effects. The other groups consisted of 158 (61 with baseline data) patients treated by radiotherapy alone, 272 (146 with baseline data) patients who had treatment with chemotherapy alone and 81 (46 with baseline data) patients who had received both chemotherapy and radiotherapy at some stage of their treatment.

The majority of patients in the radiotherapy group received dogleg radiotherapy to a dose of 30 Gy for stage I seminoma. The majority of patients treated by chemotherapy received cisplatin-based chemotherapy, most commonly with the BEP regimen (44%), but a third of patients received a carboplatin-based regimen (carboplatin alone or carboplatin/etoposide/bleomycin). Patients in the combined group most commonly (70%) received radiotherapy after chemotherapy for advanced disease, although approximately 10% of these patients had received single agent carboplatin and radiotherapy for stage IIa/b seminoma (Table 2).

### Fertility

Only 207 (30%) patients reported attempting conception after treatment. In the surveillance group, 34% of patients conceived children following diagnosis compared to 18–21% of patients in groups receiving treatment (Table 3). Some caution has to be used in interpreting these data as they reflect both the level of fertility and how commonly patients in each group attempted conception. Only 22% of the radiotherapy group, who have a higher median age than the other groups, attempted conception compared to 39% in the surveillance group.

When attempted, the success rate was high with 159 (77%) reporting success without intervention and a further 10 patients (5%) were successful after infertility treatment (four by artificial insemination and six by *in vitro* fertilisation). There was a trend to lower success rate in groups receiving chemotherapy (S (85% success without fertility treatment), any chemotherapy 70% *P*=0.028).

### FSH

There was evidence of significant elevations of FSH in all groups (Table 4). In total, 42% of surveillance patients had an FSH above the normal range (>10 iu l^−1^). Elevated FSH was found more frequently in all groups of patients receiving treatment, with the combination treatment group (70% elevated) being the most frequently affected (Table 4).

Patients with elevated FSH were significantly less likely to have conceived children after their treatment. In total, 55 of 311 (17.7%) patients with elevated FSH (68% of the 81 who tried to conceive) had children compared to 101 of 329 (30.7%) with normal FSH levels (91% of 111 who tried to conceive) (*P*<0.001). In contrast, there was no difference in the conception rate of patients with abnormal compared to normal testosterone levels.

### LH/testosterone

This survey revealed significant gonadal dysfunction in all patient groups. These changes are summarised in Table 4. In the surveillance group, 11% of patients had a subnormal level of testosterone (<10 nmol l^−1^) and 6% had evidence of a raised LH (>12 iu l^−1^). Treatment with chemotherapy or radiotherapy alone resulted in mean levels of LH higher than after orchidectomy alone (*P*<0.01), but this did not affect testosterone levels. Patients who received both chemotherapy and radiotherapy, however, did have significantly greater risk of low testosterone levels (*P*=0.006). Hypogonadism was common in this group with 38% of patients having either subnormal testosterone or testosterone replacement.

### Comparison of hormonal function at baseline and at follow-up

Data at baseline and follow-up are available on 367 patients. These results are shown in Table 5. The median levels of their hormones at follow-up of this group are similar to the levels for the group as a whole (Table 4). At baseline, patients receiving chemotherapy had significantly lower FSH levels and higher LH and testosterone levels. There was a weak correlation between the level of HCG and LH levels, suggesting a degree of interaction between the two factors (*R*=0.266, *P*<0.001). There was also a similarly weak correlation between age and FSH levels (*R*=0.264, *P*<0.0001), and testosterone (*R*=0.247, *P*<0.0001), but not for LH. However, these correlations only partially explain this difference.

On follow–up, FSH levels tend to be higher than at baseline. Treatment with chemotherapy (alone or in combination with radiotherapy) resulted in both higher FSH levels (despite lower baseline levels) and greater rises in FSH over time.

Patients on surveillance had 1.0 nmol l^−1^ rise in median testosterone compared to baseline levels on follow–up, but after chemotherapy there was a fall in testosterone (−2 nmol l^−1^) (*P*<0.01). As baseline levels for chemotherapy patients were initially higher, the end result was of similar testosterone levels at follow-up with or without chemotherapy except in the combination treatment group (*P*=0.02).

### Sexuality and treatment

**Figure 1:**
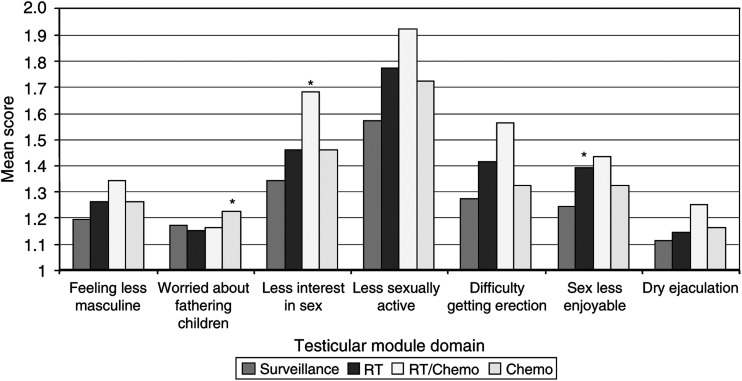
Effect of treatment level on sexual quality of life. Mean scores represent the average score for the question. Each question is scored on a 4-point scale from 1 (none) to 4 (very much). Results statistically significant different (*P*⩽0.05) from surveillance are indicated by ^*^.

We have investigated the long-term effects of treatment on sexuality by using the testicular module of the EORTC Qly C 30. This consists of six questions directed at sexual function and sexual satisfaction and two additional questions about effects on masculinity and concerns about fathering children. The results of these questions have been analysed by comparing the overall scores of patient groups and by the number of patients reporting significant effect (‘quite a bit’ or ‘very much’). A proportion of patients (between 60 and 165 of 680 patients) did not answer these questions with no significant bias by treatment group. Overall sexual function seemed to be satisfactory with 83% of patients expressing satisfaction in their sexual relationships with their partner with no differences between treatment groups (Figure 1). Compared to surveillance there was a tendency for treated groups to have less sexual activity and less interest in sex. This was only statistically significant for CRT and less interest in sex (*P*=0.01) and borderline significant for CRT and RT and less sexual activity (both *P*=0.051). Additionally, radiotherapy treatment was associated with reduced sexual enjoyment (RT *vs* S *P*=0.05). Compared to surveillance patients, patients treated by chemotherapy had more worries about fathering children (*P*=0.009).

### Effects of hormonal function on patients' quality of life

**Figure 2:**
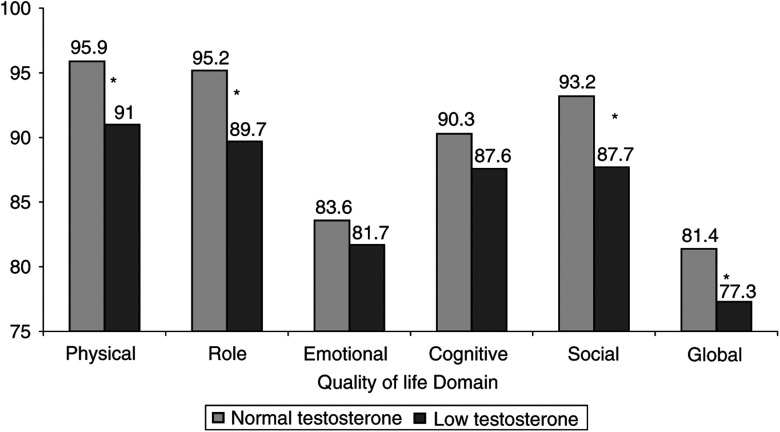
Effect of hormone levels on quality of life. Mean scores represent the average score for the question. Each question is scored on a 4-point scale from 1 (none) to 4 (very much). Results statistically significant different (*P*⩽0.05) from surveillance are indicated by ^*^.

Although 72% of patients with low testosterone continued to report that sex was satisfying, there was evidence of significant morbidity in all six domains of the testicular quality of life module that relate to sexual activity. These results are summarised in Table 6. In addition to effects on sexuality, men with a low testosterone had evidence of more general impairment in quality of life. Low testosterone levels were associated with lower quality of life scores for physical (*P*<0.001), social (*P*=0.002) and role functioning (*P*=0.003) and a trend to impaired global QoL (*P*=0.05) (Figure 2). Low testosterone was also associated with a marked increase in dyspnoea (mean score 14.3 (95% CI 9.4–19.3) compared to 6.4 (CI 5.0–7.8) *P*<0.001) and lesser increases in the complaint of pain (*P*=0.048), sleep disturbance (*P*=0.039), constipation (*P*=0.03) and nausea (*P*=0.031).

Patients with elevated LH levels reported more worries regarding fathering a child (*P*=0.001), less sexual activity (*P*=0.005), but otherwise there were no significant associations with reduced sexual quality of life. Elevated FSH levels were not associated with impaired sexual quality of life apart from feelings of less masculinity, which is of borderline significance (*P*=0.018).

**Figure 3:**
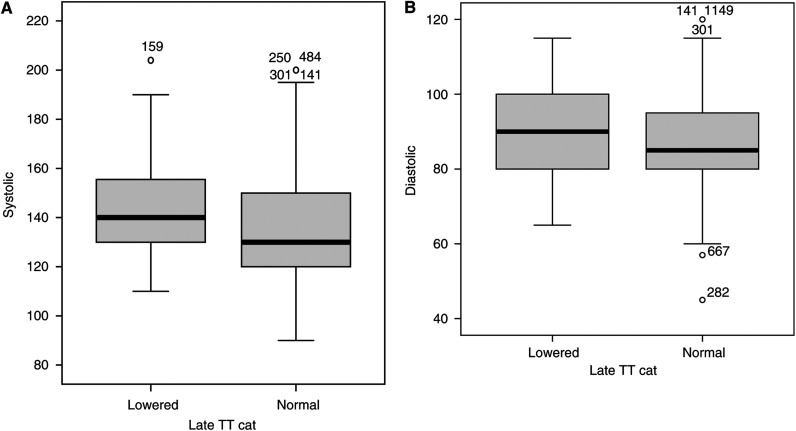
Relationship between systolic (**A**) and diastolic (**B**) blood pressure and testosterone status. Lowered refers to a testosterone level below the normal range (see text for details).

As well as quality of life effects, we have previously recognised that low testosterone is associated with a higher body mass Index compared to those with normal testosterone levels (median BMI of 28.6 compared to 25.7 kg m^−2^; *P*<0.001) (Huddart and Norman, 2003). Low testosterone is also associated with higher systolic (median 140 compared to 130 *P*<0.004) and diastolic blood pressure (median 90 compared to mean 85 *P*<0.016) (Figure 3).

## DISCUSSION

The development of testicular cancers is associated with other testicular abnormalities such as testicular maldescent, testicular atrophy and subfertility (United Kingdom Testicular Cancer Study Group, 1994; Moller and Skakkebaek, 1999; Fossa and Kravdal, 2000). Preorchidectomy sperm counts and LH levels are lower and FSH levels higher than age-matched normal controls and patients presenting with lymphoma in a Danish study (Petersen *et al*, 1999). Initial management in most patients is with orchidectomy, so it could be anticipated there would be evidence of gonadal dysfunction in a significant proportion of patients. In this study we have confirmed that indeed gonadal dysfunction is common after treatment for testicular cancer and it is of some concern that we note that over 13% of our patients have either a subnormal testosterone or are on testosterone replacement therapy. There are only limited published data on the hormonal function of patients after orchidectomy alone (summarised in Table 7). The two largest previous studies reported findings similar to ours with subnormal testosterone in 5% (Gerl *et al*, 2001) and 16% (Aass *et al*, 1991) of patients.

The deleterious effects of low testosterone are well recognised in a number of patient groups including patients receiving hormone therapy for prostate cancer (Bates *et al*, 1996; Simon *et al*, 1997; Smith *et al*, 2001). Our results clearly show a similar impact on the quality of life of testicular cancer patients with low testosterone. We have already demonstrated in a previous report that low testosterone is associated with increases in body mass index (Huddart and Norman, 2003) and that elevated LH and FSH are associated on univariate (though not multivariate) analysis with an increased risk of cardiac events (Huddart *et al*, 2003). Additionally, in this study we have shown that patients with low testosterone have higher average systolic and diastolic blood pressure. As would be anticipated, we have shown that a low testosterone has a significant effect on the quality of sexual experience, although it is interesting to observe that up to three quarters of men with low testosterone report satisfaction with their sexual life. We do not have direct data on how much this represents satisfaction with their relationships rather than sexual activity *per se* or whether lower libido allows satisfaction with lower activity. A similar effect on sexual activity of mild leydig cell insufficiency has also been reported in men after treatment for haematological malignancy (Howell *et al*, 2000). Low testosterone was also associated with a reduction in more general aspects on quality of life including physical functioning, role and social effects. The difference between patients with normal and low testosterone in these domains is in the order of 5–6%. The clinical significance of these differences is open to interpretation. It is commonly considered that a difference of 10% on these scales is an important difference for an individual patient. In these averaged results, which contain patients with lesser and greater differences, we believe these statistically significant results represent important and clinically relevant findings. We can only speculate on whether this is a direct effect or secondary to effects on sexual functioning and masculinity. Fatigue was not significantly worse in these patients with a low testosterone, a finding similar to that of Howell *et al* (2000) in haematological patients, but, perhaps surprisingly, patients with a low testosterone reported high levels of dyspnoea. The reason for this is unclear.

In addition to low testosterone, we have also noted elevations of LH and FSH in a significant proportion of our patients, a finding similar to other smaller studies (Hansen *et al*, 1990; Stuart *et al*, 1990; Gerl *et al*, 2001). A recent large Norwegian study found similar abnormalities in LH, which were significantly elevated compared to normal controls even in those treated by orchidectomy alone (Nord *et al*, 2003).

In common with other reports we have observed increases in LH and FSH levels following chemotherapy (Table 4). However, the level of gonadal dysfunction due to the treatment effect seems to be on average less than that reported in previous studies (Table 7). There may be a number of reasons for this. A number of studies have shown that the gonadal dysfunction is greatest immediately after chemotherapy (Stuart *et al*, 1990; Brennemann *et al*, 1997, 1998). For instance, Brennemann *et al* (1997) in a cross-sectional study showed a higher rate of elevated LH and FSH levels in the first year compared to the number measured after 8 years of follow-up (LH 32% *vs* 3.6%, FSH 89% *vs* 64%). As the median follow-up of our cohort is over 10 years, this lower level of dysfunction may reflect that a degree of recovery of the pituitary–gonadal axis has occurred with time.

However, this may not be the complete explanation as Gerl *et al* (2001) could detect no difference in LH, FSH and testosterone levels in patients measured between 24 and 60 months and over 60 months. Our Longitudinal data suggest that for surveillance patients there is little change or even a small rise in levels with follow-up. Seeing a rise in median levels would be unexpected as testosterone levels usually decline with age, but could be explained by temporary reduction in testosterone at diagnosis due to tumour development or the effect of recent orchidectomy, which recovers with follow-up. This contrasts with chemotherapy-treated patients, who have higher baseline levels of testosterone and LH and lower FSH levels at diagnosis. This may be due to an interaction between LH and HCG levels and their younger median age (for FSH and testosterone). On follow-up, on average, both testosterone and LH levels fell after treatment. For most patients this fall is modest, but 25% of patients had a greater than 8 ng ml^−1^ fall in testosterone. As there is difference in baseline values between those who received chemotherapy and patients managed by surveillance, the net effect is to achieve levels similar to that seen in patients treated by orchidectomy alone. The data on testosterone could suggest an effect of chemotherapy on testosterone levels, but could be equally interpreted as being due to a differential effect on baseline results due to disease or age-related factors.

Our study could also differ from previous reports due to differences in the chemotherapy utilised. Previous studies have suggested greater dysfunction in patients receiving higher doses of cisplatin chemotherapy (Berger *et al*, 1996; Bokemeyer *et al*, 1996; Gerl *et al*, 1997), vinblastine (Berger *et al*, 1996; Bokemeyer *et al*, 1996) and ifosphamide (Brennemann *et al*, 1997). The majority of our chemotherapy-treated patients received etoposide rather than ifosphamide- or vinblastine-based chemotherapy and additionally almost a third of our patients received carboplatin rather than cisplatin. This view is supported by the greater degree of hormonal dysfunction seen in our intensively treated combination treatment group, many of whom had received intensive salvage treatment.

A number of studies have reported the effects of direct testicular irradiation when applied for carcinoma *in situ*, but there is little long-term data on the effect of scattered irradiation from an abdominal field (Fossa *et al*, 1993; Brennemann *et al*, 1997; Petersen *et al*, 2002). Despite statistically significant higher LH levels, our results, like the previous studies, suggest that dogleg radiotherapy probably results in minimal long-term hormonal effects.

In this study we have not undertaken analysis of spermatogenesis, but a number of studies (Fosså *et al*, 1985; Kreuser *et al*, 1989; Aass *et al*, 1991; Brennemann *et al*, 1998; Jacobsen *et al*, 2001) have emphasised the close correlation between FSH levels and sperm counts. On this basis the elevated level of FSH would suggest that there is a significant long-term impairment of spermatogenesis. In support of this view we were able to observe a clear relationship between FSH elevation and the ability to conceive. However, this is not a clearcut relationship as many patients with elevated FSH do still remain fertile. Our results have shown that though success is less likely with a raised FSH, over 2/3 of such patients were still able to conceive.

The long-term effect of chemotherapy on spermatogenesis at our institution has been previously reported (Lampe *et al*, 1995). In this study we noted that 80% of patients who were normospermic before treatment had spermatogenesis by 5 years and the chance of recovery was better if carboplatin and less than four cycles were used. Similar results have been reported by other authors (Drasga *et al*, 1983; Fossa *et al*, 1993; Petersen *et al*, 1994). For instance, Drasga *et al* (1983) found that 46% of patients had normal sperm counts 2–3 years after treatment despite only 6.6% having normal spermatogenesis pretreatment. The results of our previous study are reflected in the gratifying success rate of patients attempting to have children despite the substantial evidence of gonadal dysfunction. Although only 25% of patients had successful conception, this represented 77% of the 207 patients who reported attempting conception. This rose to 82% with the aid of infertility treatment. Radiotherapy treatment had no detrimental effect on this success rate, although only a small proportion of patients attempted conception. This is in keeping with the observations in the MRC-randomised trial of dogleg *vs* para-aortic radiotherapy that by 3 years 92% of patients treated by dogleg radiotherapy have re-attained sperm counts of 10 million ml^−1^ or greater.

Success rate appeared to be lower after chemotherapy, in line with the elevations in FSH that we observed. Most of this is likely to be a direct effect on germ cell function, but there may also be a contribution from dry ejaculation which was reported as ‘quite a bit’ or ‘very much’ in 3% or more patients after chemotherapy compared to surveillance (Figure 1). We do not have direct data comparing fertility rates of our patients with normal controls, but in a study of Norwegian registry data (Fossa and Kravdal, 2000) fertility was approximately 30% lower in testicular cancer patients than the normal population. In line with our findings they reported that lower rates were seen in men who had regional and distant disease (who presumably had more intensive treatment) (fertility rate 0.49 (CI 0.36–0.64) compared to 1.0 for normal population) rather than patients with local disease (0.85 CI 0.67–1.06).

The frequency of hypogonadism observed suggests that screening for testicular dysfunction should be a routine part of testicular cancer follow-up. However, careful thought will need to be given to the issue as to how one manages any detected hypogonadism. Hormone supplementation is indicated for the symptomatic patient with obvious potency problems. The correct management approach for the ‘asymptomatic’ man with reasonable sexual activity is more difficult. Testosterone can be replaced by a number of routes (e.g. patches, gels, implanted pellets), but still the most common are by intramuscular injections, which when commenced are likely to be lifelong. Data from other sources have suggested that hypogonadism is associated with the development of osteoporosis and other health effects. Our data have highlighted the, perhaps more subtle, effects of hypogonadism, which affects cardiac risk factors such as BMI and blood pressure and the range of quality of life effects. It is likely that decisions on this will have to be individualised after careful discussion of the issues, but suggests that replacement therapy needs to be considered for this patient group.

In summary, we have found that hormonal dysfunction is frequent after the diagnosis of testicular cancer and treatment can have an additional detrimental effect. This can have a significant impact on the quality of life and contribute to other health problems. Most patients, however, remain fertile though this can be affected by their treatment. Screening for hormonal problems should be considered as a routine part of patient management.

## Figures and Tables

**Table 1:** Characteristics of patients in long-term follow-up study

	**No. of patients assessed**	**Chemotherapy**	**Chemotherapy/ radiotherapy**	**Radiotherapy**	**Surveillance**	**Total**
*Patients in cohort*						
All patients treated 1982–1992		660	271	331	341	1603
Full data available		292	90	175	182	739
Patients eligible for fertility study$		272	81	158	169	680
						
*Number of deaths*						
Dead of TT		67	74	0^a^	0^a^	141
Dead of other causes		24	10	16	12	62
Age at presentation (median and range)	1603	28 (10–62)	32 (15–68)	35 (19–82)	31 (13–82)	31.71 (10–82)
Age at follow-up (median and range)	739	41 (23–72)	47 (30–69)	47 (29–78)	45 (27–76)	44 (23–78)
Median follow-up (years)	1603	9.7 years (0–19.8)	11.9 years (0.2–19.9)	9.48 years (0.1–20.3)	11.4 years (0–19.9)	10.2 (0–20.3)

a All patients relapsing after radiotherapy or surveillance will have received chemotherapy so will be included in one of the other groups and hence there are no deaths of disease in these groups.

$ To be eligible for this study, patients had to have one or more remaining testicles.

**Table 2:** Disease and treatment characteristics of patients treated at The Royal Marsden Hospital between 1982 and 1992 entered into this study

	**Chemotherapy**	**Chemotherapy/radiotherapy**	**Radiotherapy**	**Surveillance**	**Overall**
*N*	272	81	158	169	680
*Histology*					
Seminoma	32	46	157	57	292
NSGCT	240	35	1	112	388
					
a *Chemotherapy regimes*					
Platinum	120	19			139
BEP					
BOP	35	3			38
PVB	12	14			26
Other platinum regimes	13	3			16
Carboplatin					
Only	18	33			51
JEB	74	7			87
Other	0	2			2
					
*Reason for radiotherapy*					
Elective after chemotherapy		61	—		61
Stage I seminoma		4	130		134
Stage II seminoma		12	27		39
Stage I/II NSGCT		4	1		5

NSGCT, nonseminomatous germ cell tumour.

a B, Bleomycin; E, Etoposide; P, Cisplatinum; J, Carboplatin; V, vinblastine; O, vincristine.

**Table 3:** Summary of the ability to conceive after treatment for testicular cancer

	**Chemotherapy**	**Chemotherapy/radiotherapy**	**Radiotherapy**	**Surveillance**	**Total**
*N*	272	81	158	169	680
Tried to conceive	83 (31%)	24 (30%)	34 (22%)	66 (39%)	207 (30%)
Successful (no infertility treatment)	59 (71%)	16 (67%)	28 (82%)	56 (85%)	159 (77%)
Successful with infertility treatment	3	4	1	2	10
Unsuccessful	21	4	5	8	38
Overall success rate^a^	75%	83%	85%	88%	82%

a Successful with or without infertility treatment out of all who tried to conceive.

**Table 4:** Hormonal levels in patients on long-term follow-up by treatment group

	**Chemotherapy**	**Chemotherapy radiotherapy**	**Radiotherapy**	**Surveillance**
*Testosterone*				
Mean	14.8	12.8	14	14.6
Median	14	12^**^	14	14%
Low	13%	34%	15%	11%
Testosterone replacement	0%	4%	0.60%	0
				
*FSH*				
Mean	14.6	21.5	13.8	11.9
Median	10^*^	19^***^	10	9
Raised	49%	71.00%	45%	41%
				
*LH*				
Mean	7.5	9.8	7.2	5.8
Median	6^***^	8^***^	6^**^	5
Raised	10%	22%	11%	6%

Raised or low levels indicate above or below the normal ranges for assay (testosterone 10–30 ng ml^−1^, FSH 1–10 iu l^−1^, LH 1–12 iu l^−1^). Asterisks indicate significant difference to surveillance patients (^*^P<0.05, ^**^P<0.01, ^***^P<0.001).

**Table 5:** Hormonal levels in patients with paired baseline and follow-up hormone profiles

	**Chemotherapy**	**Chemotherapy/radiotherapy**	**Radiotherapy**	**Surveillance**	**Overall**
*N*	146	46	61	114	367
*FSH (iu 1^−1^)*					
Baseline	4* (2–8)	8 (3.75–19)	7 (5–10)	7 (4–10)	6
Follow-up	10* (8–18)	19* (9.25–31.75)	10 (7–15.25)	9 (6–13.25)	10
Difference	6* (3–9.25)	11* (4–19)	3 (1–7)	3 (1–5)	4
					
*LH (iu 1^−1^)*					
Baseline	11* (6–38.75)	9* (6–17.25)	8 (5–10)	6 (4–10)	8
Follow-up	6* (4–9)	8* (5–12)	6* (4–8)	5 (4–7)	6
Difference	–4* (−28.5 to −1)	−1 (−5.5–4.5)	−1 (−3–1.0)	−1 (−4–1.0)	−2
					
*Testosterone (nmol l^−1^)*					
Baseline	17* (13–21)	13 (10–16)	13 (11–16)	13 (11–17)	15
Follow-up	14 (11–17.5)	12 (9–17)	14 (10–16)	14 (11–17)	14
Difference	−2* (−8–1.5)	−2 (−5.25–4.0)	−1 (−4–2.0)	1 (−4–4.0)	−1

All baseline levels are postorchidectomy and prechemotherapy or radiotherapy. Follow-up samples are a minimum of 5 years post-treatment. The presented data are not normally distributed, so presented values in the table are median (interquartile range). Normal ranges as presented in Table 4.

* Statistically different to surveillance (*P*<0.01).

**Table 6:** Effect of testosterone on sexual quality of life

		**Testosterone level**		
**Category**	***n***	**Normal**	**Low**	
Worried about fathering child	622	22/502 (4%)	11/95 (12%)	*P*=0.01
Less interested in sex	600	45/486 (9%)	22/92 (24%)	*P*<0.001
Less sexually active	599	97/484 (20%)	33/93 (35%)	*P*=0.001
Sex less enjoyable	550	25/449 (6%)	14/81 (17%)	*P*<0.001
Difficulty getting erection	553	28/481 (6%)	12/81 (14%)	*P*=0.008
Sex has been satisfying	515	358/420 (85%)	53/74 (72%)	*P*=0.004
Feeling less masculine	624	19/504 (4%)	9/95 (9%)	*P*=0.03

Data are presented as the proportion of patients reporting ‘quite a bit’ or ‘very much’ for the question. Comparisons between groups undertaken by Fishers exact test.

**Table 7:** Overview of data on hormonal function of long term survivors of testicular cancer

	**Proportion of patients with abnormal hormone levels**							
	**Orchidectomy only**				**Orchidectomy plus chemotherapy**			
**Publication**	***n***	**Testosterone**	**LH**	**FSH**	***n***	**Testosterone (%)**	**LH (%)**	**FSH (%)**
Drasga *et al* (1983)	—	—	—	—	28	25	43	53
Leitner *et al* (1986)	—	—	—	—	22	0	86	63*
Kreuser *et al* (1989)	—	—	—	—	44	0	2	10
Stuart *et al* (1990)	11	0	45%	45%	27	0	67	75
Hansen *et al* (1990)	9	0	11%	NS	22	13*	59*	86
Aass *et al* (1991)	36	16%	6%	24%	42	14	37	37*
Berger *et al* (1996)	—	—	—	—	63	10	24	63
Brennemann *et al* (1997)	11	NS	NS	27%	232	5.2	13.4	64
Gerl *et al* (2001)	58	5%	12%	53%	117	11	19	53
Jacobsen *et al* (2001)	60	NS	NS	17%	—	—	—	—
Strumberger *et al* (2001)	—	—	—	—	32	12	47	75
Huddart *et al* (this series)	179	13%	8.7%	43%	290	15	13*	50*

* Significantly different from result with no chemotherapy. NS=not stated.

## References

[bib1] Aass N, Fossa SD, Theodorsen L, Norman N (1991) Prediction of long-term gonadal toxicity after standard treatment for testicular cancer. Eur J Cancer 27: 1087–1091 1720322 10.1016/0277-5379(91)90298-r

[bib2] Bates AS, Van't Hoff W, Jones PJ, Clayton RN (1996) The effect of hypopituitarism on life expectancy. J Clin Endocrinol Metab 81: 1169–1172 8772595 10.1210/jcem.81.3.8772595

[bib3] Berger CC, Bokemeyer C, Schuppert F, Schmoll HJ (1996) Endocrinological late effects after chemotherapy for testicular cancer. Br J Cancer 73: 1108–1114 8624272 10.1038/bjc.1996.213 PMC2074412

[bib4] Bokemeyer C, Berger CC, Kuczyk MA, Schmoll HJ (1996) Evaluation of long-term toxicity after chemotherapy for testicular cancer. J Clin Oncol 14: 2923–2932 8918489 10.1200/JCO.1996.14.11.2923

[bib5] Brennemann W, Stoffel-Wagner B, Helmers A, Mezger J, Jager N, Klingmuller D (1997) Gonadal function of patients treated with cisplatin based chemotherapy for germ cell cancer. J Urol 158: 844–850 9258096 10.1097/00005392-199709000-00041

[bib6] Brennemann W, Stoffel-Wagner B, Wichers M, Helmers A, Albers P, Mezger J, Klingmuller D (1998) Pretreatment follicle-stimulating hormone: a prognostic serum marker of spermatogenesis status in patients treated for germ cell cancer. J Urol 159: 1942–1946 9598493 10.1016/S0022-5347(01)63203-8

[bib7] Drasga RE, Einhorn LH, Williams SD, Patel DN, Stevens EE (1983) Fertility after chemotherapy for testicular cancer. J Clin Oncol 1: 179–183 6199473 10.1200/JCO.1983.1.3.179

[bib8] Fayers P, Aaronson N, Bjordal K, Groenvold M, VCurran D, Bottomley A, Group, o.b.o.t.E.Q.o.L (2001) The EORTC QLQ-C30 Scoring Manual EORTC Quality of Life Group, Brussels. European Organisation for Research and Treatment of Cancer, Brussels

[bib9] Fossa SD, Aabyholm T, Vespestad S, Norman N, Ous S (1993) Semen quality after treatment for testicular cancer. Eur Urol 23: 172–176 8477777 10.1159/000474589

[bib10] Fossa SD, Kravdal O (2000) Fertility in Norwegian testicular cancer patients. Br J Cancer 82: 737–741 10682691 10.1054/bjoc.1999.0989 PMC2363334

[bib11] Fosså SD, Ous S, Abyholm T, Loeb M (1985) (I) Post-treatment fertility in patients with testicular cancer. I. Influence of retroperitoneal lymph node dissection on ejaculatory potency. Br J Urol 57: 204–209 2580586 10.1111/j.1464-410x.1985.tb06425.x

[bib12] Gerl A, Clemm C, Schmeller N, Hentrich M, Lamerz R, Wilmanns W (1997) Late relapse of germ cell tumors after cisplatin-based chemotherapy. Ann Oncol 8: 41–47 10.1023/a:1008253323854 9093706

[bib13] Gerl A, Muhlbayer D, Hansmann G, Mraz W, Hiddemann W (2001) The impact of chemotherapy on Leydig cell function in long term survivors of germ cell tumors. Cancer 91: 1297–1303 11283930 10.1002/1097-0142(20010401)91:7<1297::aid-cncr1132>3.0.co;2-z

[bib14] Hansen SW, Berthelsen JG, von der Maase H (1990) Long-term fertility and Leydig cell function in patients treated for germ cell cancer with cisplatin, vinblastine, and bleomycin *vs* surveillance. J Clin Oncol 8: 1695–1698 1698935 10.1200/JCO.1990.8.10.1695

[bib15] Howell SJ, Radford JA, Smets EM, Shalet SM (2000) Fatigue, sexual function and mood following treatment for haematological malignancy: the impact of mild Leydig cell dysfunction. Br J Cancer 82: 789–793 10732747 10.1054/bjoc.1999.1000 PMC2374403

[bib16] Huddart R, Norman A, Shahidi M, Horwich A, Coward D, Nicholls E, Dearnley D (2003) Cardiovascular disease as a long-term complication of treatment for testicular cancer. J Clin Oncol 21: 1513–1523 12697875 10.1200/JCO.2003.04.173

[bib17] Huddart RA, Norman A (2003) Changes in BMI after treatment of testicular cancer are due to age and hormonal function and not chemotherapy. Br J Cancer 89: 1143–1144; author reply 1145 12966440 10.1038/sj.bjc.6601178 PMC2376940

[bib18] Jacobsen KD, Theodorsen L, Fossa SD (2001) Spermatogenesis after unilateral orchiectomy for testicular cancer in patients following surveillance policy. J Urol 165: 93–96 11125372 10.1097/00005392-200101000-00023

[bib19] Kreuser ED, Kurrle E, Hetzel WD, Heymer B, Porzsolt F, Hautmann R, Gaus W, Schlipf U, Pfeiffer EF, Heimpel H (1989) Reversible germ cell toxicity following aggressive chemotherapy in patients with testicular tumors: results of a prospective study. Klin Wochenschr 67: 367–378 2501552 10.1007/BF01711264

[bib20] Lampe H, Dearnaley DP, Price A, Mehta J, Powles R, Nicholls J, Horwich A (1995) High-dose carboplatin and etoposide for salvage chemotherapy of germ cell tumours. Eur J Cancer 5: 717–723 10.1016/0959-8049(95)00018-e 7640044

[bib21] Leitner SP, Bosl GJ, Bajorunas D (1986) Gonadal dysfunction in patients treated for metastatic germ-cell tumors. J Clin Oncol 4(10): 1500–1505 3020183 10.1200/JCO.1986.4.10.1500

[bib22] Moller H, Skakkebaek N (1999) Risk of testicular cancer in subfertile men: case–control study. BMJ 318: 559–562 10037628 10.1136/bmj.318.7183.559 PMC27753

[bib23] Nord C, Bjoro T, Ellingsen D, Mykletun A, Dahl O, Klepp O, Bremnes RM, Wist E, Fossa SD (2003) Gonadal hormones in long-term survivors 10 years after treatment for unilateral testicular cancer. Eur Urol 44: 322–328 12932930 10.1016/s0302-2838(03)00263-x

[bib24] Petersen PM, Giwercman A, Daugaard G, Rorth M, Petersen JH, Skakkeaek NE, Hansen SW, von der Maase H (2002) Effect of graded testicular doses of radiotherapy in patients treated for carcinoma-*in-situ* in the testis. J Clin Oncol 20: 1537–1543 11896102 10.1200/JCO.2002.20.6.1537

[bib25] Petersen PM, Hansen SW, Giwercman A, Rorth M, Skakkebaek NE (1994) Dose-dependent impairment of testicular function in patients treated with cisplatin-based chemotherapy for germ cell cancer. Ann Oncol 5: 355–358 8075033 10.1093/oxfordjournals.annonc.a058840

[bib26] Petersen PM, Skakkebaek NE, Vistisen K, Rorth M, Giwercman A (1999) Semen quality and reproductive hormones before orchiectomy in men with testicular cancer. J Clin Oncol 17: 941–947 10071288 10.1200/JCO.1999.17.3.941

[bib27] Simon D, Charles MA, Nahoul K, Orssaud G, Kremski J, Hully V, Joubert E, Papoz L, Eschwege E (1997) Association between plasma total testosterone and cardiovascular risk factors in healthy adult men: The Telecom Study. J Clin Endocrinol Metab 82: 682–685 9024276 10.1210/jcem.82.2.3766

[bib28] Smith JC, Bennett S, Evans LM, Kynaston HG, Parmar M, Mason MD, Cockcroft JR, Scanlon MF, Davies JS (2001) The effects of induced hypogonadism on arterial stiffness, body composition, and metabolic parameters in males with prostate cancer. J Clin Endocrinol Metab 86: 4261–4267 11549659 10.1210/jcem.86.9.7851

[bib29] Strumberg D, Brugge S, Korn MW, Koeppen S, Ranft J, Scheiber G, Reiners C, Mockel C, Seeber S, Scheulen ME (2002) Evaluation of long-term toxicity in patients after cisplatin-based chemotherapy for non-seminomatous testicular cancer. Ann Oncol 13(2): 229–236 11885999 10.1093/annonc/mdf058

[bib30] Stuart NS, Woodroffe CM, Grundy R, Cullen MH (1990) Long-term toxicity of chemotherapy for testicular cancer – the cost of cure. Br J Cancer 61: 479–484 2109631 10.1038/bjc.1990.106 PMC1971302

[bib31] United Kingdom Testicular Cancer Study Group (1994) Aetiology of testicular cancer; association with congenital abnormalities, age at puberty, infertility, and exercise. BMJ 308: 1393–1399 7912596 PMC2540340

